# Microstructural changes in CoCrFeMnNi under mild tribological load

**DOI:** 10.1007/s10853-020-04806-0

**Published:** 2020-05-27

**Authors:** Antje Dollmann, Alexander Kauffmann, Martin Heilmaier, Christian Haug, Christian Greiner

**Affiliations:** 1 Institute for Applied Materials (IAM), Karlsruhe Institute of Technology (KIT), Kaiserstrasse 12, 76131 Karlsruhe, Germany; 2 KIT IAM-CMS MicroTribology Center (µTC), Strasse am Forum 5, 76131 Karlsruhe, Germany

## Abstract

The lack of a principle element in high-entropy alloys (HEA) leads to unique and unexpected material properties. Tribological loading of metallic materials often results in deformed subsurface layers. As the microstructure feedbacks with friction forces, the microstructural evolution is highly dynamic and complex. The concept of HEAs promises high solid solution strengthening, which might decrease these microstructural changes. Here, we experimentally investigated the deformation behavior of CoCrFeMnNi in a dry, reciprocating tribological contact under a mild normal load. After only a single stroke, a surprisingly thick subsurface deformation layer was observed. This layer is characterized by nanocrystalline grains, twins and bands of localized dislocation motion. Twinning was found to be decisive for the overall thickness of this layer, and twin formation within the stress field of the moving sphere is analyzed. The localization of dislocation activity, caused by planar slip, results in a grain rotation. Fragmentation of twins and dislocation rearrangement lead to a nanocrystalline layer underneath the worn surface. In addition, oxide-rich layers were found after several sliding cycles. These oxides intermix with the nanocrystalline layer due to material transfer to the counter body and re-deposition to the wear track. Having revealed these fundamental mechanisms, the evolution of such deformation layers in CoCrFeMnNi under a tribological load might lead to other HEAs with compositions and properties specifically tailored to tribological applications in the future.

**Electronic supplementary material:**

The online version of this article (10.1007/s10853-020-04806-0) contains supplementary material, which is available to authorized users.

## Introduction

Tribology, the study of friction, wear and lubrication, has accompanied mankind for thousands of years. Friction is influenced by the complex combination of parameters such as the materials constituting the tribological contact, their surface roughnesses, the normal force, sliding speed, ambient media and possible lubrication [1]. For metallic materials, tribological loading leads to microstructural changes in the subsurface area [2–4]. These changes themselves feedback with the stress field and friction properties of the tribological system itself [5]. Tribological loading of coarse grained materials is known to result in crystal rotation [3, 6], twinning [7], the formation of band-like patterns [8, 9], subgrains [3, 6], and nanocrystalline layers [5, 10] as well as phase transformations [8]. Several material systems have been investigated in the literature, such as copper [3, 6, 10–12], steels [7, 8, 13], Cu–Ni–Sn bronze [9], nickel [14] and gold [5], to only name a few.

It was proposed that solid solutions—such as high-entropy alloys (HEAs)—with high concentration of several solute atoms exhibit improved tribological properties due to an increased strength and corrosion resistance [15]. Hence, HEAs which consist of at least five elements in almost equiatomic proportion and obtain a single-phase microstructure may be promising materials for tribological applications [15].

In terms of their application in friction contacts, the influence of their exact composition [16], different lubricants [17] and manufacturing routes (e.g., casting [18], laser cladding [19] or spark plasma sintering [20]) was studied. The self-lubrication properties of HEAs were tested by adding graphite, MoS_2_, silver powder or soft dispersoids [21, 22]. This holds also for precipitation strengthening by borides [23], carbides [24], nitrides [25] or by allowing for complex, as-cast microstructures [18]. Typically, the coefficient of friction and an analysis of the wear mechanisms are reported. Scanning electron microscopy (SEM) of the wear track is often utilized and the wear volume is identified, e.g., by profilometry [17]. It was reported that the wear resistance of HEAs can be higher than that of steel [26], Co-based Stellite®6 [24] and Ti–6Al–4V [27].

Instead of being involved in development of materials with complex multi-phase microstructures for friction applications, we are rather interested in revealing the deformation mechanisms active under a tribological load from just a single trace up to thousands of sliding cycles [3, 11, 12]. In this context, the question arose whether the compositional complexity of high-entropy alloys also translates in an equally complex deformation behavior under the shear loading imposed by a friction contact. This issue is not yet sufficiently investigated in the existing literature. To address it, we have chosen the well-known single-phase, face centered cubic (fcc) CoCrFeMnNi alloy. As this material is solid solution strengthened [15], dislocation movement and interactions are expected to be mainly influenced by properties inherent to concentrated solid solutions like lattice distortion and also by fundamental parameters of deformation in fcc metals, e.g., stacking fault energy (SFE). The focus of our study is on changes in the subsurface microstructure. We hypothesize fewer subsurface changes when compared to copper [3, 6] based on the strain hardening behavior of CoCrFeMnNi [28]. This strain hardening of CoCrFeMnNi is due to the medium SFE between 18 and 27 mJ m^−2^ [29], even leading to twinning induced plasticity (TWIP) [30].

As severe adhesive wear was reported when sliding against Si_3_N_4_ [17], we paired CoCrFeMnNi against sapphire spheres and deliberately chose mild loading conditions. Especially performing experiments with a small number of reciprocating cycles—down to only a single trace—allows to analyze the onset of microstructural changes and to follow the development of these deformation layers. With an enhanced knowledge about these microstructural changes and their origin, alloys and surface structures can effectively be tailored to improve tribological properties in the future, thereby allowing to conserve energy and resources.

## Materials and methods

Elemental bulk materials were used for manufacturing CoCrFeMnNi samples. Co, Cr, Fe, Mn and Ni with purities of 99.95%, 99+%, 99.99%, 99.99% and 99.97%, respectively, were mixed according to their stoichiometric composition. The elements were melted in Ar atmosphere, using an AM/0.5 arc melting furnace (Edmund Bühler GmbH, Bodelshausen, Germany). To maintain the purity of the final material, Mn was etched in a solution of water and nitric acid in a ratio of 10:1 prior to melting. The melting chamber was pumped to a pressure of 5 · 10^–2^ mbar three times and subsequently flooded with Ar before pumping to a high vacuum of less than 1 · 10^–4^ mbar. Once more, the chamber was flooded with Ar to a base pressure of 600 mbar. Prior to each melting step, a Zr getter was liquefied to further decrease the amount of residual oxygen. The initial buttons were flipped and re-melted at least four times. Finally, the CoCrFeMnNi samples were cast in a rectangular-shaped, water-cooled Cu mold with the dimensions (2.0 · 5.0 · 1.5) cm^3^. Subsequently, the samples were homogenized at 1200 °C for 72 h in evacuated fused silica tubes and immediately quenched in water. The homogenized samples were rolled from a thickness of 15 mm down to 5 mm and recrystallized at 1000 °C in an evacuated tube for 1 h. The heat treatment was finished by quenching in water. The initial grain size was 38.7 µm as determined from linear intercepts in backscatter electron images. The exact composition of the alloy was determined via inductively coupled plasma optical emission spectrometry (ICP-OES) to 15.2 at.% Co, 21.7 at.% Cr, 21.2 at.% Fe, 21.2 at.% Mn and 20.1 at.% Ni (accuracy of 0.1 at.%).

Prior to tribological testing, the samples were ground with SiC paper down to grit P4000 and mechanically polished using 3 µm and 1 µm diamond suspension for at least 8 min each (DP-suspensions M products from Struers, Stuttgart, Germany). For the following electropolishing, an electrolyte of perchloric acid and methanol with a ratio of 1:9 was used. This preparation process ensures a deformation layer free surface, see Figure S1 in the Supplementary Information.

For the dry sliding experiments, a reciprocating linear tribometer was used with CoCrFeMnNi paired against 10-mm-diameter sapphire spheres (Saphirwerk, Brügg, Switzerland). To investigate the influence of increasing cycle numbers, tests with 0.5, one, five, ten, 100, 500 and 1000 cycles were conducted. Hereby, one cycle is defined as a forward and backward movement, meaning that the counterbody was only moved in one direction for 0.5 cycles. Each wear track was performed at a new sample location and with a new sphere. All other experimental parameters were held constant: a normal force of 2 N, sliding speed of 0.5 mm s^−1^, stroke length of 12 mm, room temperature (25 °C) and a relative humidity of 50% ± 3%. The corresponding Hertzian contact pressure [31] was 655 MPa, calculated with a Young’s modulus of 172 GPa for CoCrFeMnNi and 430 GPa for sapphire, the Poisson ratio was 0.3 for both materials.

Microstructural changes were examined by using a dual beam focused ion beam (FIB), scanning electron microscope (Helios NanoLab™ DualBeam™ 650 from ThermoFisher Scientific, Hillsboro, USA). Two platinum layers were deposited to protect the surface from ion beam damage. The electron beam was used for depositing the first layer and the focused ion beam for the thicker second layer. Cross sections were cut with the focused ion beam, and secondary electron (SE) pictures were taken with an acceleration voltage of 2 kV and a current of 0.8 nA. For further microstructural investigations, transmission electron microscopy (TEM) foils were prepared with a state of the art FIB lift-out technique with little ion beam damage [32]. Scanning transmission electron microscopy (STEM) bright field (BF) images were taken with an acceleration voltage of 30 kV and a current of 100 pA for the electron beam in the before mentioned dual beam microscope.

The TEM foils were analyzed by cross-sectional electron backscattered diffraction (CS-EBSD) [14] and for higher resolution with transmission Kikuchi diffraction (TKD). The scans were performed on a pre-tilted surface of 70° and 20°, respectively, with an acceleration voltage of 30 kV and a beam current of 6.4 nA. The step size was 20 nm for CS-EBSD and 10 nm for TKD, respectively. The Kikuchi patterns were recorded with a NordlysMax^2^ detector and indexed by the AZtecHKL software (both, Oxford Instruments, Abingdon-on-Thames, UK). Further analysis was performed with the Matlab toolbox MTEX [33]. Beside maps color-coded according to the inverse pole figure of the normal direction (ND), Kernel average misorientation (KAM) maps were also calculated. For the Kernel calculations, the first-order neighbors are used with a maximum misorientation of 1°. Energy-dispersive X-ray spectroscopy (EDS) scans were conducted using an X-Max 80 detector (Oxford Instruments, Abingdon-on-Thames, UK) at an acceleration voltage of 20 kV and beam current of 6.4 nA.

## Results

### Friction and wear mechanisms

**Figure 1:**
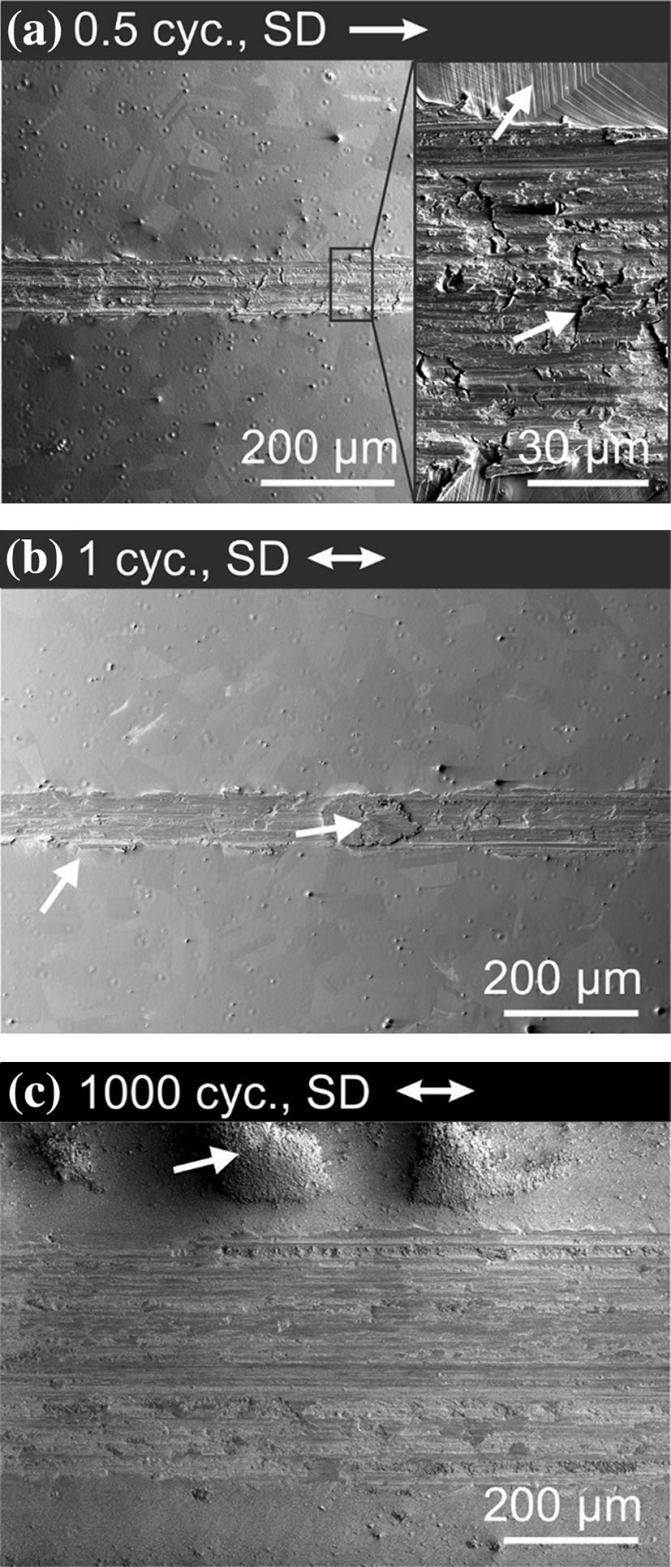
Scanning electron microscopy images of wear tracks for different cycle numbers. **a** 0.5 cycles, the inset shows a higher magnified image of the wear track; **b** after one cycle; and **c** after 1000 cycles. The white arrows above the images indicate the sliding direction (SD). The arrows in the images indicate flakes in (**a**), slip traces in (**a**) and (**b**), re-deposited material in (**b**) and wear particles in (**c**), respectively

The worn surfaces after 0.5, one and 1000 cycles were studied by electron microscopy as presented in Fig. 1. The inset in Fig. 1a shows a higher magnification of the wear track. Slip traces are seen next to the wear track as indicated by a white arrow. The wear track’s surface shows flakes marked by a white arrow and grooves in sliding direction (SD). Similar features occurred after one cycle (Fig. 1b). Additionally, a spot with an oval shape is found as indicated by a white arrow. After 1000 cycles (Fig. 1c), wear particles appear next to the wear track, while fewer flakes are observed.

**Figure 2:**
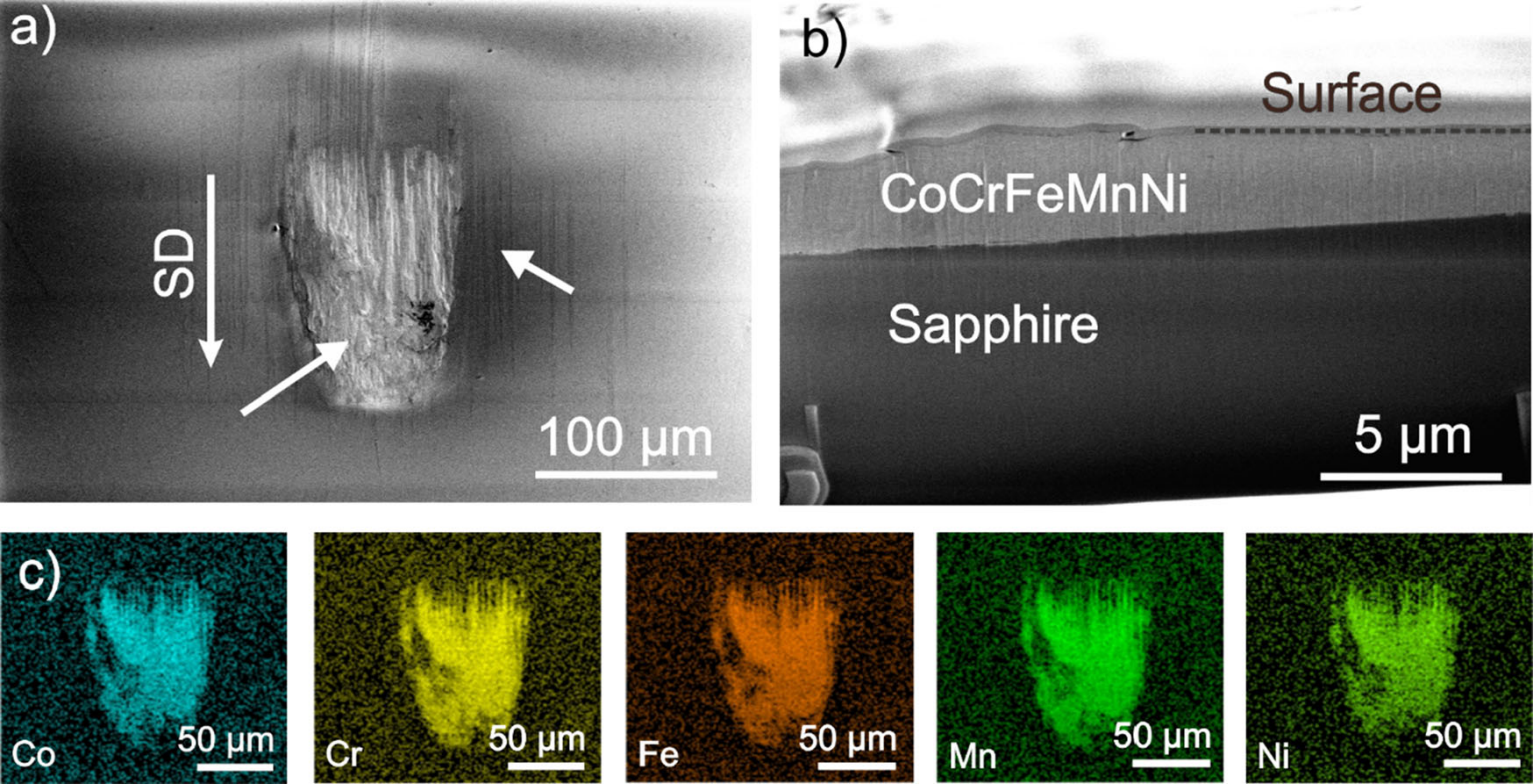
Top and cross-sectional images of the counter body after 0.5 cycles and corresponding EDS mapping. **a** Top view; **b** cross-sectional view; and **c** EDS measurement of the area shown in (**a**) with Co, Cr, Fe, Mn and Ni mappings. The sliding direction (SD) is indicated by an arrow in (**a**). The white arrows indicate material transfer and grooves in (**a**). The dashed line in (**b**) marks the interface between the sample surface and the deposited Pt-layers for the cutting process

As tribology is always a matter of two bodies in contact, Fig. 2a presents an SEM image of the contact area of the sapphire sphere after 0.5 cycles. The center of this image shows an inhomogeneity. At this position, the FIB cross section presented in Fig. 2b reveals the existence of a layer of CoCrFeMnNi which was transferred to the sphere. This observation is confirmed by the identification of Co, Cr, Fe, Mn and Ni by EDS (Fig. 2c). Next to the adhesive CoCrFeNiMn (Fig. 2a, left white arrow), grooves in SD (right white arrow) have been formed.

**Figure 3:**
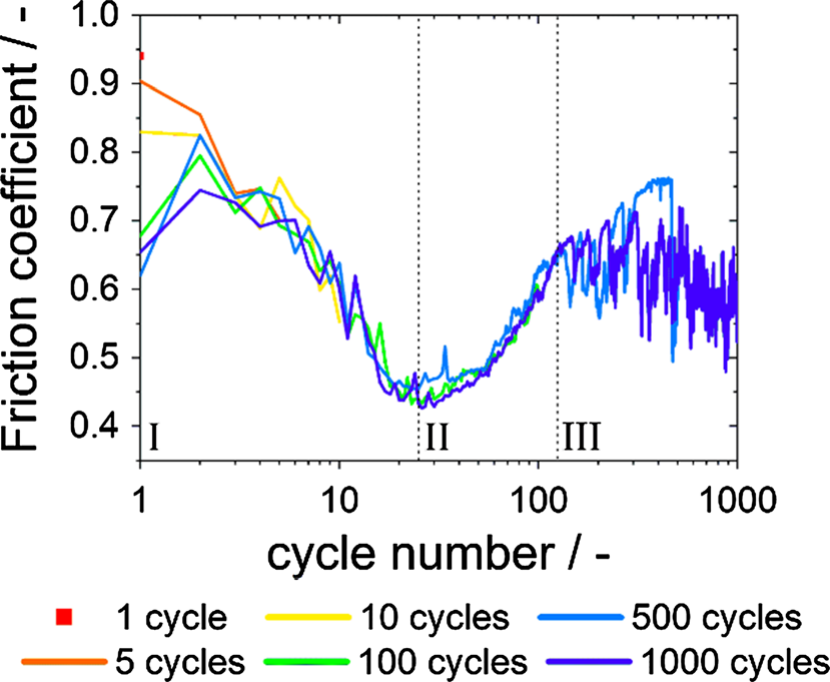
Friction coefficient as a function of cycle number. Unlubricated reciprocating sliding of sapphire spheres on CoCrFeMnNi for 1, 5, 10, 100, 500 and 1000 cycles. The abscissa is logarithmic. The plot is divided in the regions: I, II and III

The coefficient of friction (*µ*) is plotted as a function of cycle number in Fig. 3. To ease the comparison between experiments conducted with varying number of sliding cycles (one, five, ten, 100, 500, 1000 cycles), a logarithmic abscissa is chosen. While the friction coefficient for very low cycle numbers is roughly between 0.6 and 0.9, *µ* follows similar characteristics after the first seven cycles. The friction behavior as a function of cycle number can be divided in three characteristic regions marked by Roman numerals in Fig. 3: (I) a decrease in *µ* for up to 25 cycles, (II) an increase in *µ* for up to around 120 cycles and (III) a plateau of *µ* at about 0.65 for up to 1000 cycles.

### Microstructural evolution

**Figure 4:**
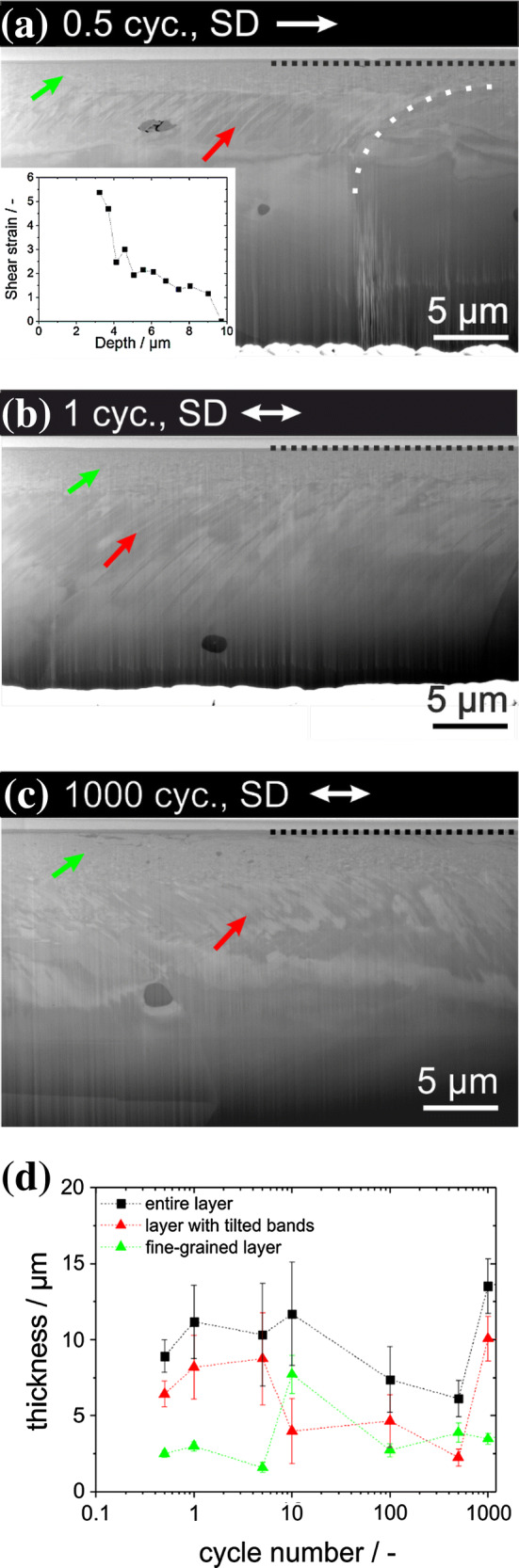
Scanning electron microscopy images of cross sections with increasing cycle numbers. **a** after 0.5 cycles, the inset shows the shear strain depending on the distance from the surface, calculated from the bent grain boundary marked by a white, dashed line; **b** after one cycle; and **c** after 1000 cycles. The cross sections were prepared in the center of the wear track, along the sliding direction (SD), shown by the white arrow. The dashed lines mark the interface between the sample surface and the deposited Pt-layers for the cutting process. The green arrows indicate the fine-grained layer and the red arrows the layer with tilted bands. **d** Deformation layer thicknesses for the fine-grained layer, the layer with tilted bands and the entire layer as a function of the cycle number are given. The dashed lines are to guide the eyes. The observed particles in **a**–**c** are oxides, which might originate from the manufacturing route

In order to evaluate the evolution of the subsurface microstructure, FIB cross sections were cut parallel to the SD and in the center of the wear tracks after all experiments. For example, the cross sections for the wear tracks after 0.5, one and 1000 cycles are presented in Fig. 4a–c. In Fig. 4a, the deformation layer can be divided into two zones of different characteristics. The layer close to the surface appears to be fine-grained (green arrow). The second layer, being located between the fine-grained layer and the bulk material, is identified by bands tilted in SD (red arrow). Additionally, a preexisting grain boundary is severely bent in SD and is tagged with a dashed white line in Fig. 4a. Using this bending of the grain boundary, we calculated the shear strain at the surface using a routine described elsewhere [9, 12]. The shear strain is *γ* = d*x*/d*y*, with d*x* being the decay length in SD and d*y* the distance from the surface to the boundary in ND. The shear strain profile is presented as an inset in Fig. 4a. Very close to the surface—down to about 1 µm, the shear strain could not be calculated as the original grain boundary was no longer visible there. The material after one and 1000 cycles exhibits a similar sequence of deformation layers as the one after 0.5 cycles: A fine-grained top layer and a layer of tilted band-like features underneath (Fig. 4b, c). In Fig. 4d, the thicknesses of the fine-grained and the layer with tilted bands as well as the total layer thickness are plotted as a function of the cycle number; the latter plotted in a logarithmic manner. The total deformation layer thickness is between 8.9 ± 1.1 µm after 0.5 cycles and 14.4 ± 2.3 µm after 1000 cycles. Exceptions occur at 100 and at 500 cycles, where the layers are even thinner than after 0.5 cycles. For the vast majority of samples analyzed, the fine-grained layer assumes half of the thickness of the layer with tilted bands, with notable exceptions after ten and 500 cycles. Note that the layers are only referred to as fine-grained and with tilted bands when describing the cross-sectional scanning electron microscopy images.

**Figure 5:**
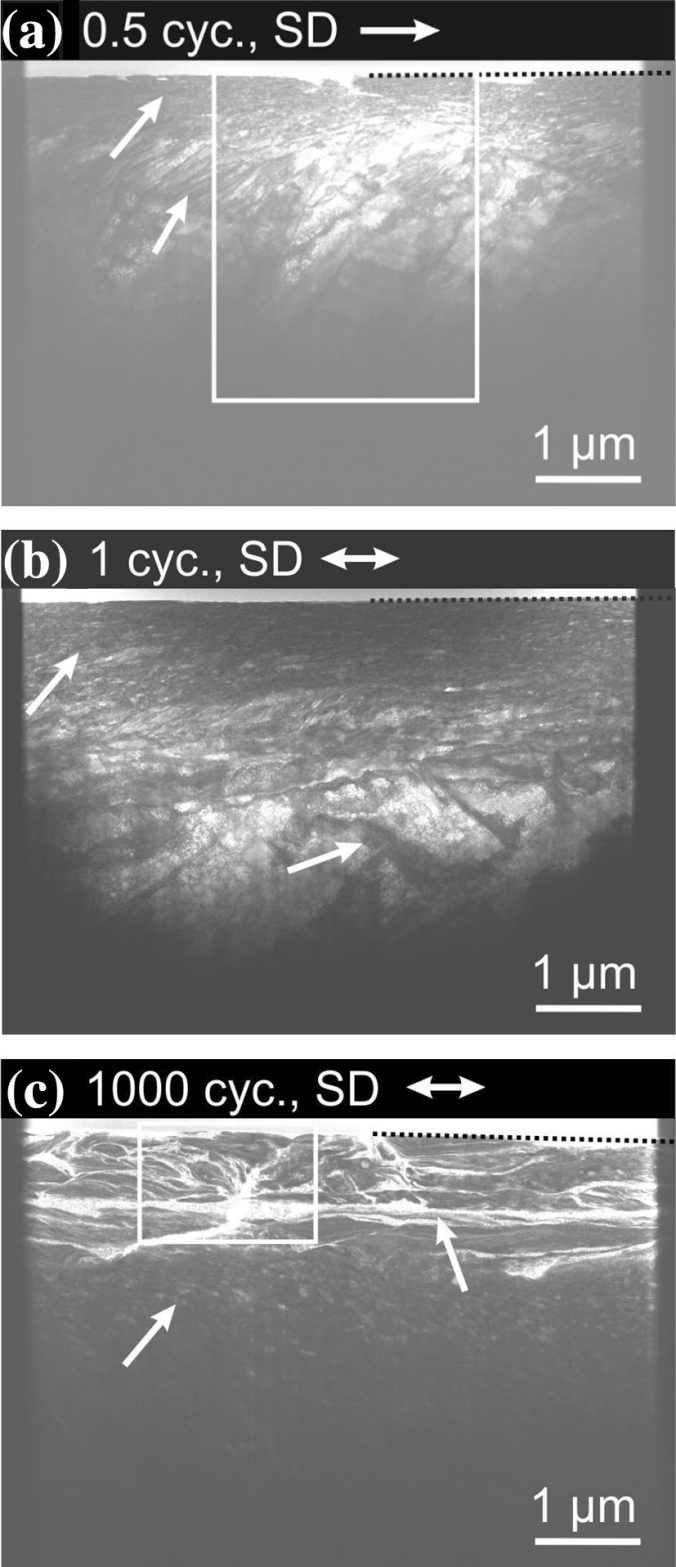
Transmission scanning electron microscopy images for increasing cycle numbers. **a** After 0.5 cycles; **b** after one cycle; and **c** after 1000 cycles. The TEM-foils were prepared in the center of the wear track, along the sliding direction (SD) (shown as the white arrow in the headline). The dashed lines mark the interface between the sample surface and the deposited Pt-layers for the cutting process. The arrows in the images indicate nanocrystalline grains in (**a**), (**b**) and (**c**), respectively; tilted bands in (**a**) and (**b**); bands with bright contrast in (**c**). The rectangles in (**a**) and (**c**) indicate the regions of the TKD scans in Fig. 6 and the EDS scans in Fig. 11, respectively

For more detailed microstructure investigations, TEM foils were prepared after 0.5, one and 1000 cycles, again parallel to SD and in the center of the wear track. The corresponding STEM bright field images are shown in Fig. 5. Beneath the surface, nano-sized grains (indicated by a white arrow) are detected in all the depicted deformation layers after 0.5, one and 1000 cycles. In the STEM images after 0.5 and one cycle (Fig. 5a, b), bands marked with a white arrow are visible with different lengths, widths, distances and tilt angles with respect to the SD. With increasing depth, the bands are longer, broader and exhibit a greater spacing. A crack at the worn surface is visible in Fig. 5a. After 1000 cycles (Fig. 5c), bands with bright contrast (white arrow) are observed. Their orientation is generally parallel to the surface. In the same TEM foil, a crack is found nearly perpendicular to these bright bands.

**Figure 6:**
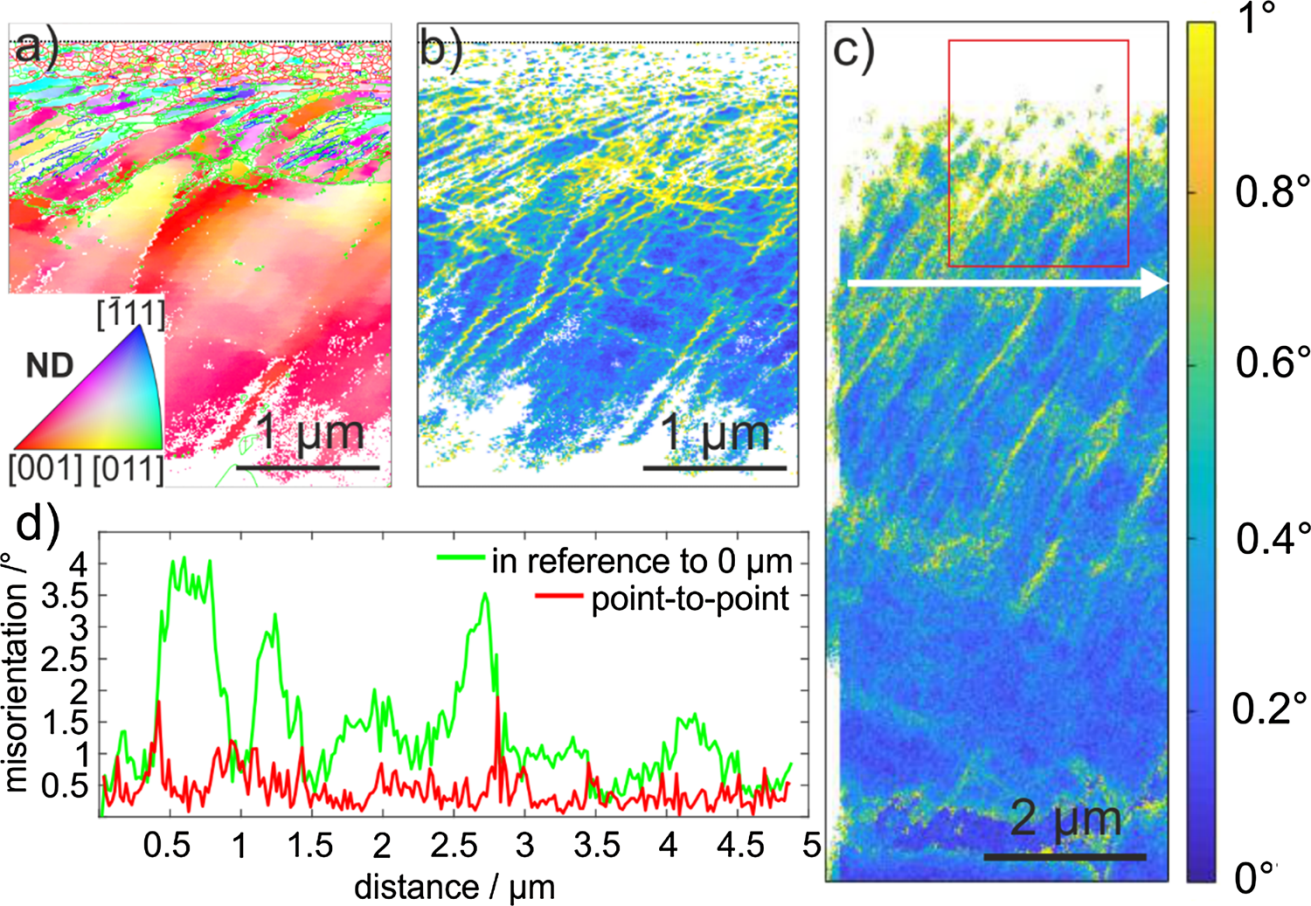
Deformation layer analysis via TKD and cross-sectional EBSD after 0.5 cycles. **a** TKD map color-coded according to the inverse pole figure of the normal direction (see the inset). Small-angle grain boundaries are highlighted in green (1°–15°), high-angle grain boundaries (> 15°) in red and twins (Σ3) in blue. **b** Corresponding KAM map of the scan in (**a**). **c** KAM map of a conventional cross-sectional EBSD scan. The red rectangle marks the region of the TKD scan. The point-to-point misorientation and misorientation in reference to the starting point along the white arrow on (**c**) is plotted in (**d**). The SD is from left to right

TKD and cross-sectional EBSD investigations were conducted for all TEM foils after 0.5 and one cycle. A TKD measurement of the area marked with a white rectangle in Fig. 5a (0.5 cycles) is presented in Fig. 6a, b. Figure 6a shows the crystallographic orientation in ND superimposed with the different types of grain boundaries. Low-angle grain boundaries up to 15° of misorientation are marked in green, high-angle grain boundaries (> 15°) in red and twins in blue (detection: Σ3 with a deviation of 5°). The sample surface is represented by a black, dashed line. Directly beneath the sliding surface, the nanocrystalline layer exhibits mainly high-angle grain boundaries. Beneath the nanocrystalline layer, twins are detected. Between the twin layer and the bulk material, a gradient in crystallographic orientation is present. When visualizing the same area in terms of Kernel average misorientation in Fig. 6b, linear features of increased local misorientation in comparison to the surrounding matrix are visible. In Fig. 6c, the CS-EBSD measurement is also presented as the Kernel average misorientation map. For this measurement, the entire TEM foil was mapped to record Kikuchi-patterns for the entire deformation layer investigated here. The position of the TKD measurement is marked by a red rectangle in Fig. 6c. Parallel to the surface in Fig. 6c, the point-to-point misorientation as well as the misorientation in reference to the starting point along the white arrow was determined and is plotted in Fig. 6d. A maximum point-to-point misorientation of 2° was detected along this line. Furthermore, there is an increase and decrease in misorientation in reference to the starting point.

**Figure 7:**
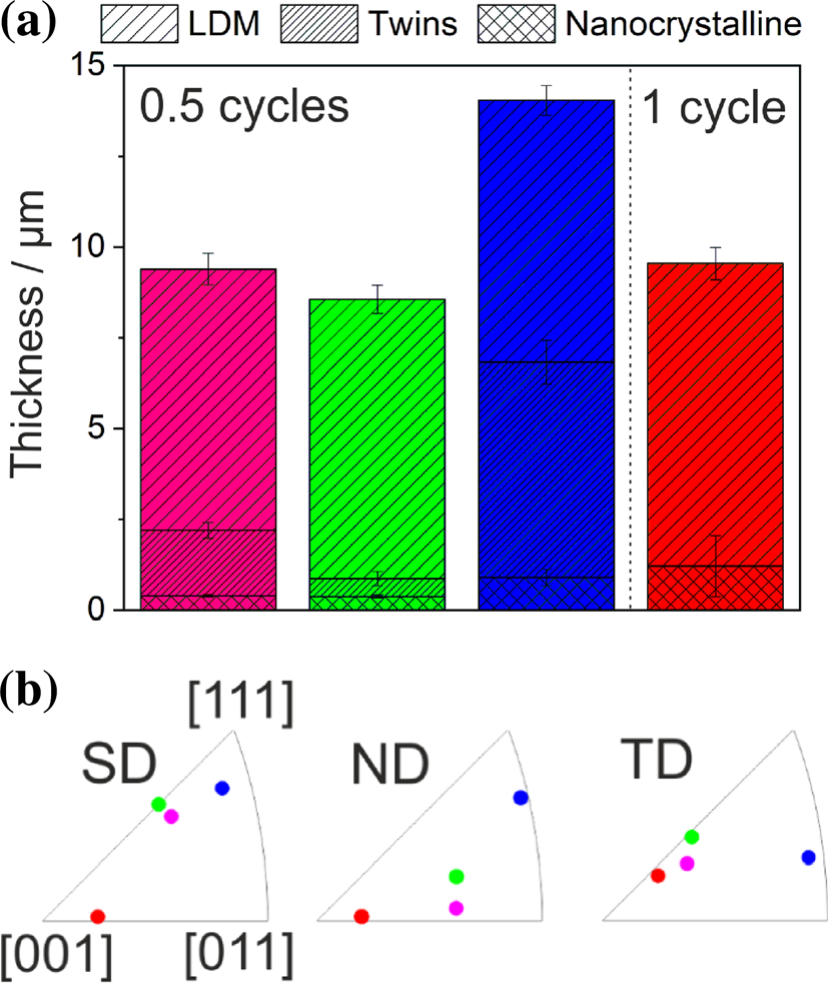
Layer thickness for varying crystallographic orientations. **a** Thicknesses of the layers containing localized dislocation movement, twins and nanocrystalline grains as well as the entire layer thickness; **b** initial crystallographic orientations presented in inverse pole figures for the sliding (SD), normal (ND) and transverse direction (TD). The pole nomenclature is equal for all directions. The color-code is the same for (**a**) and (**b**)

The maximum depth of the deformation layer as characterized by the termination of the misorientation bands was determined to be 8.2 ± 0.4 µm in Fig. 6c. The thicknesses of the nanocrystalline layer, the region with twins and the area with localized deformation were measured for three TEM foils for a wear track after 0.5 cycles. Each of these foils was cut in a grain with different crystallographic orientation in order to probe a potential orientation dependence. An additional TEM foil for a track after one cycle was analyzed, too. The results for these thicknesses are presented in Fig. 7a. The thicknesses of the layer with nanocrystalline grains and with localized deformation are almost the same for all TEM foils cut from the wear track after 0.5 cycles. After one cycle, both layer thicknesses slightly increased. A strong dependence on the crystallographic orientation is found for the twinned area’s thicknesses, varying from zero up to 5.9 ± 0.6 µm. The initial crystallographic orientation was determined for each TEM foil below the entire deformation layer in the unaffected material. Each of these orientations is represented in the inverse pole figures in Fig. 7b. The same color-code is used for all crystallographic orientations throughout this manuscript.

**Figure 8:**
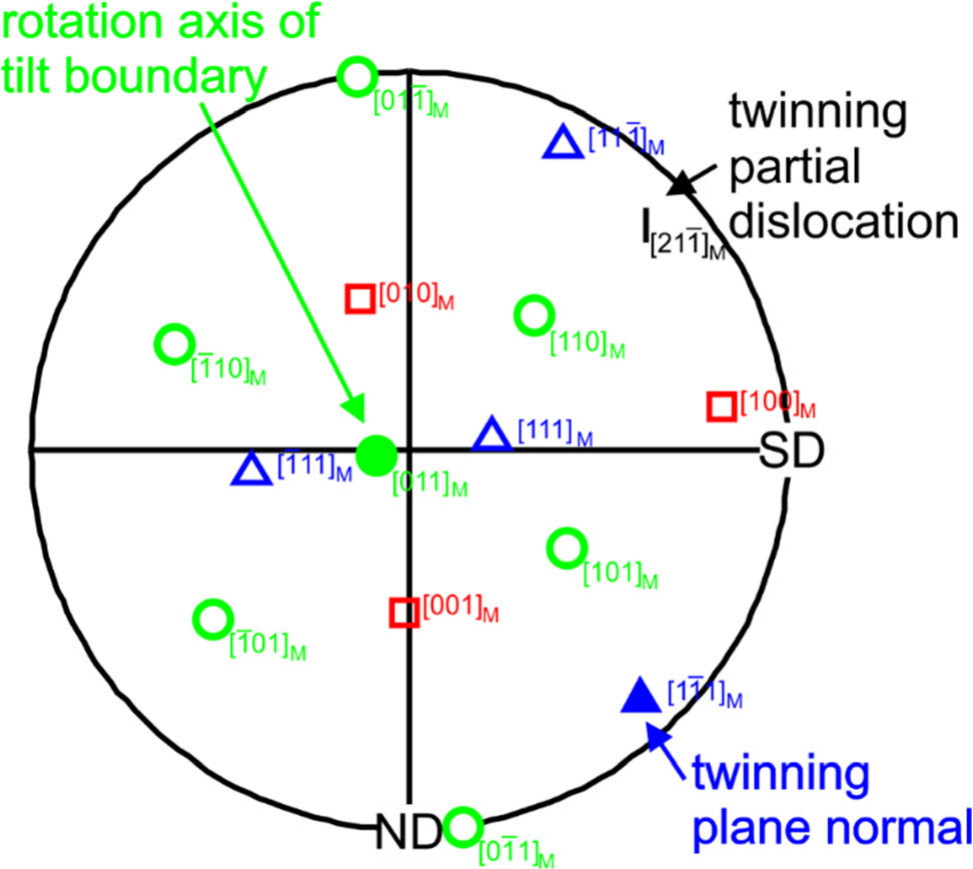
Pole figure of an analyzed twinning system. The twin of interest is marked with a black arrow in Fig. S3b. The direction of the matrix in $\langle {001} \rangle$, $\langle {011} \rangle$ and $\langle {111} \rangle$ are given, indicated with an ‘*M*.’ Each direction has its own color and open symbol. The characteristic directions such as the twinning plane normal and the rotation axis of the 70.53° $\langle {011} \rangle$ tilt boundary are marked with filled symbols. Additionally, the direction of the twinning partial dislocation is given

The twinning system within the TEM foil with the thickest twin layer (blue color-coding in Fig. 7) was further analyzed in detail. The twin of interest is marked in Figure S3b with a black arrow. The $\langle {100} \rangle$, $\langle {110} \rangle$ and $\langle {111} \rangle$ directions of the matrix next to this particular twin are given in Fig. 8. Hereby, each direction is indicated by an individual symbol color and shape. Due to the high crystal symmetry of fcc metals and alloys, the twins are both a 70.53° $\langle {110} \rangle$ tilt boundaries and a 60° $( {111} )$ twist boundaries. For the twin in question, the matrix can be rotated about the $[ {011} ]$ direction for 70.53°, resulting in the twin orientation. The same holds for a rotation around $[ {1\overline{1}1} ]$ for 60°. These two directions are marked in Fig. 8 with filled symbols. Based on these results, a Burgers vector in $[ {21\overline{1}} ]$ direction can be determined as responsible twinning dislocation and is colored in black in Fig. 8. Note, the crystal is aligned almost parallel to $[ {100} ]$ in SD and almost parallel to $[ {0\overline{1}1} ]$ in ND which is further used for the analysis of the acting stresses during loading.

**Figure 9:**
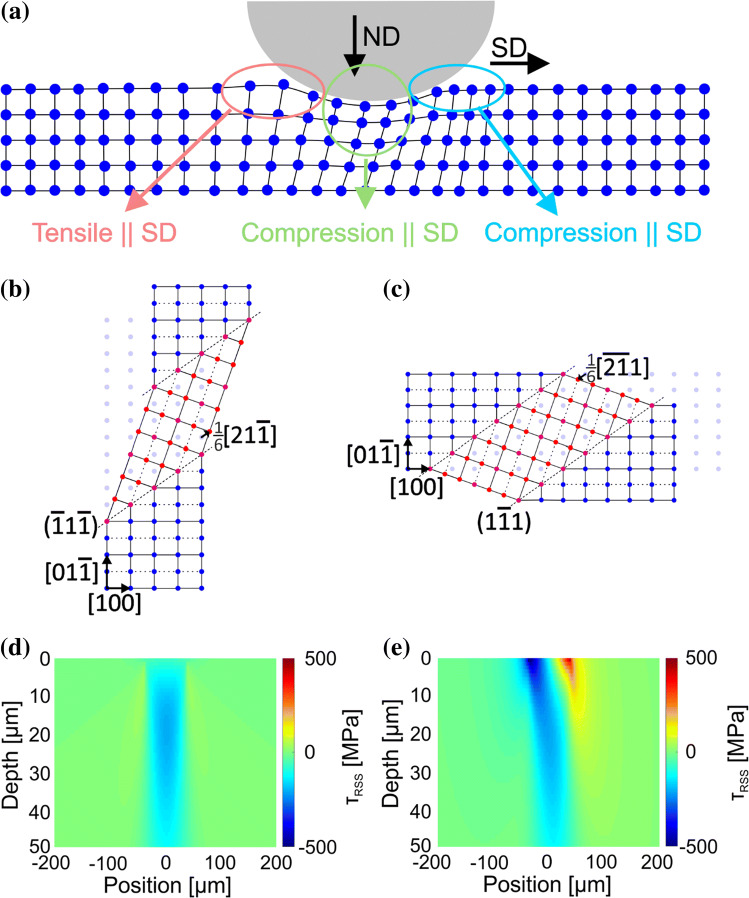
Calculation of the resolved shear stresses on the analyzed twinning system. **a** Schematic drawing of atomic displacements under a tribological load in the SD–ND plane. **b**, **c** Diagrams of geometrical possible twins for the determined twinning system in Fig. 8. Light blue dots illustrate the un-deformed crystal lattice, blue dots the matrix and red dots the atoms in the twinned area. **d** Resolved shear stress on the determined twinning system in Fig. 8 calculated after the Hertz model; **e** same as in (**d**) but with the Hamilton model, both in the SD–ND plane. The sphere is positioned at (0|0) and moves in the direction of positive position values

A schematic and simplified atomic-scale diagram visualizing the atomic displacement under tribological load in the SD–ND plane is given in Fig. 9a. Hereby, a compressive stress parallel to ND beneath the sphere, a compressive stress parallel to SD in front of the sphere and a tensile stress parallel to SD behind the sphere are illustrated. The two geometrical possibilities of the indicated twin in Fig. 8 are given in Fig. 9b, c. In the simplified cases of a uniaxial stress state, the twin in Fig. 9b would form under the tensile stress in ND or SD direction and the twin in Fig. 9c would develop under the compression stress in SD or the tensile stress in ND. The resolved shear stress (RSS) on the identified twinning system is given in Fig. 9d calculated applying the Hertzian contact model [31] and in Fig. 9e with the Hamilton solution [34], again in the SD–ND plane in both cases. Both of these models—Hertz and Hamilton—are linear elastic, whereby the Hertzian model only takes an indentation into account and the Hamilton model adds to the Hertzian model a tangential force based on the measured coefficient of friction. The sphere is located at the origin of the coordinate system used, with SD pointing in the direction of positive abscissa values.

**Figure 10:**
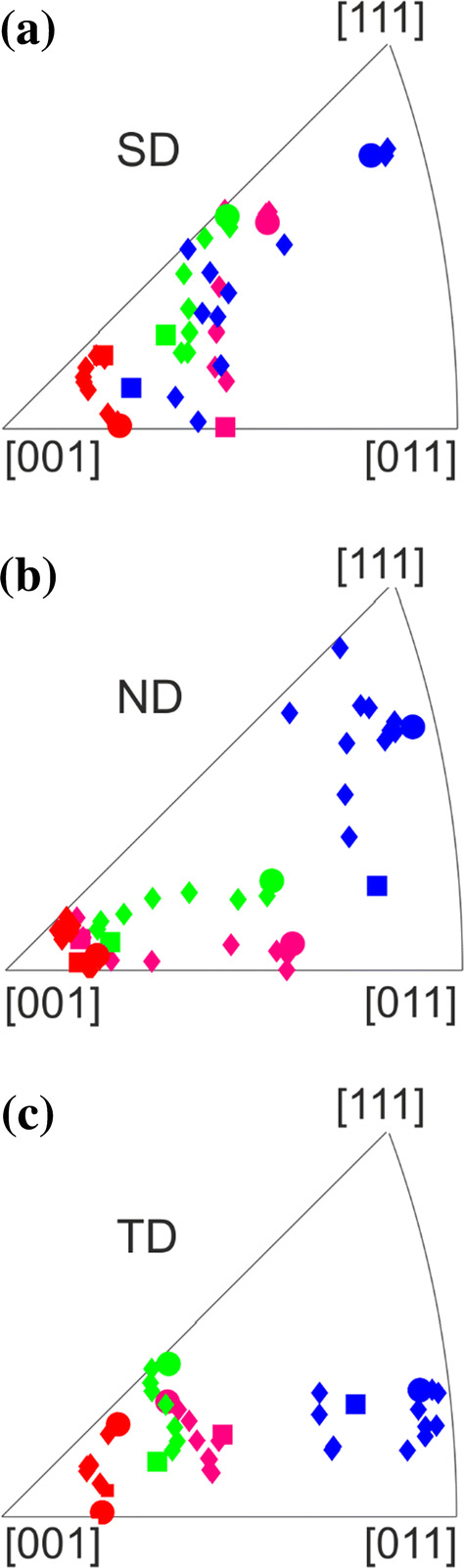
Depth-dependent texture evolution. Inverse pole figures in **a** sliding (SD); **b** normal (ND); and **c** transverse direction (TD) of averaged Euler angles from 1 µm thick layers out of the cross-sectional EBSD measurements with varying depth. Round data points represent the original crystallographic orientation and are equal to Fig. 7b. Square data points represent the layer the closest to the sample surface. Diamond data points represent the region in-between the surface and original bulk orientation

The depth-dependent crystal orientation changes in SD, ND and TD for all investigated TEM foils are plotted in Fig. 10. For calculating these orientation changes, the subsurface area of the CS-EBSD measurements (Figs. 6c, S3) was divided in rectangles of 1 µm width. In each of these rectangles, the respective Euler angles were averaged. The round data points represent the initial crystallographic orientation in Fig. 7b, whereas the square data points are the orientations closest to the surface in the CS-EBSD investigations. Diamond-shaped symbols represent the material in-between the sample surface and the initial, bulk material.

**Figure 11:**
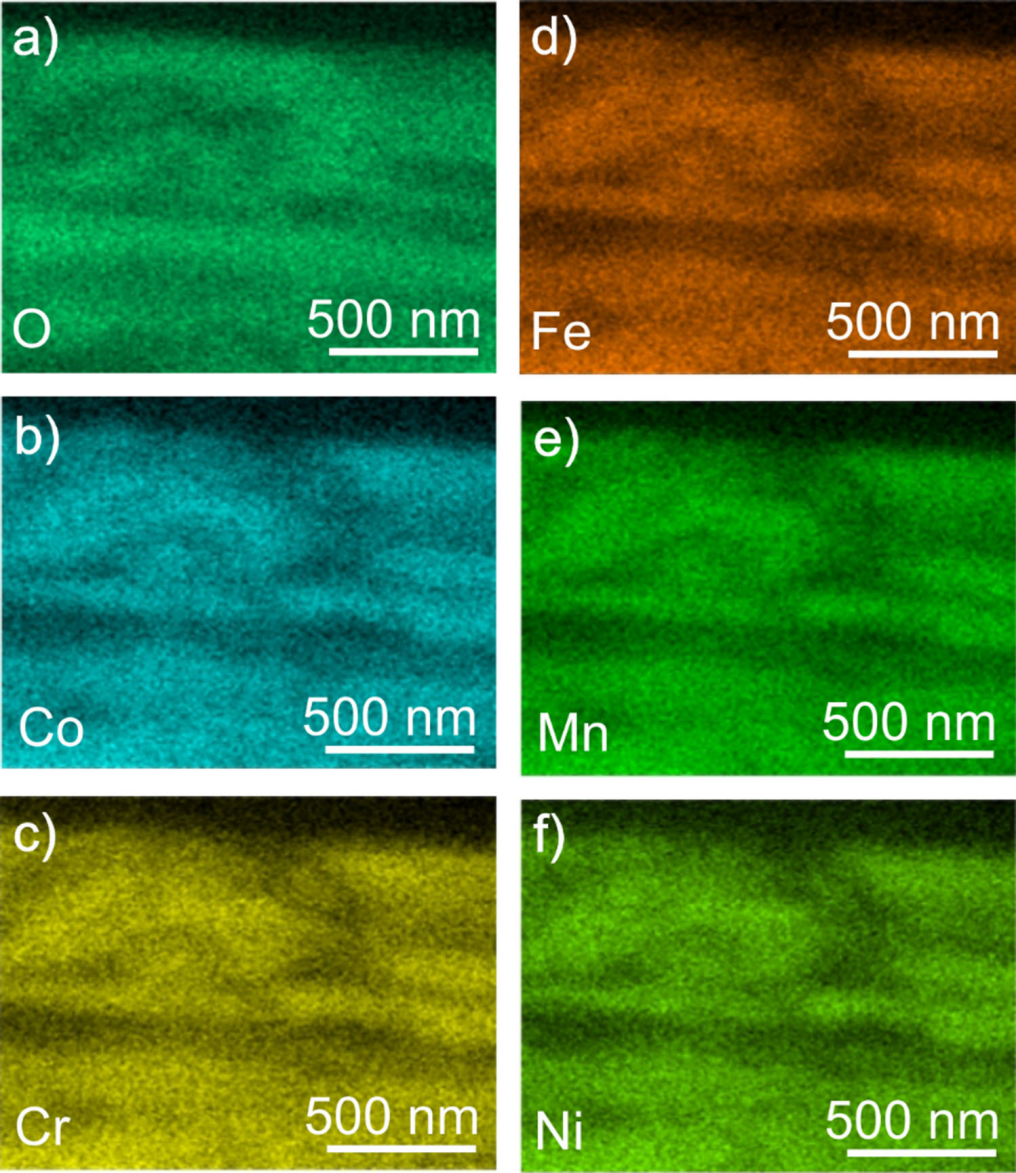
Cross-sectional EDS measurement after 1000 cycles. EDS mappings of **a** O; **b** Co; **c** Cr; **d **Fe; **e** Mn; and **f** Ni. The area of interest is shown by a white rectangle in Fig. 5c

As chemical changes commonly occur in tribologically loaded materials and to investigate the bright bands in Fig. 5c [35–37], EDS spectra were obtained in the subsurface area of a wear track after 1000 cycles (as indicated in Fig. 5c by the white rectangle). The element distribution for each of the metals constituting to CoCrFeMnNi as well as for oxygen is presented in Fig. 11. The color intensity for each element map is a qualitative representation of the elemental concentration. The figure reveals that oxygen shows an inverse intensity distribution compared to the other elements.

## Discussion

### Friction and wear mechanisms

The SEM images and EDS scans presented in Figs. 1 and 2 strongly suggest that adhesive forces acting between the HEA and the sapphire spheres led to material transfer from CoCrFeMnNi to the counter body. The worn surface of CoCrFeMnNi after 0.5 and one cycle in Fig. 1a and b shows flake-like delamination within the wear track. We speculate that material was pulled out of the surface due to strong adhesive forces. The oval-shaped region in the wear track after one cycle (Fig. 1b, upper white arrow) is most likely re-deposited CoCrFeMnNi that had been transferred to the sapphire sphere and then back to the wear track. Similar behavior for a dry contact of CoCrFeMnNi in contact with a Si_3_N_4_ sphere was reported in the literature [17], albeit for fretting conditions. An adhesive wear mechanism was also observed for other compositional complex alloys [38–40]. This being said and while there seems to be growing evidence for higher adhesive forces with HEAs, a statement about the universality of this behavior will require more and detailed studies. Material transfer changes the friction contact to a self-mated contact.

With regard to the investigations on the elementary mechanisms acting in the subsurface, it has to be considered that strong adhesive forces do not only alter the surfaces properties, but also influence the stress field acting in the subsurface material. The grooves in SD observed in the wear tracks and on the sphere (Figs. 1, 2) most likely result from oxide particles plowing through the material. These oxides might originate from the manufacturing route and are randomly distributed in the whole material, even though great care was taken to minimize possible oxidation. These oxides are also seen in the cross-sectional view in Fig. 4a–c.

The first of the three very distinct regions for the friction coefficient plotted as a function of the cycle number is characterized by the variance in initial friction coefficient and the following decrease to *µ* ≈ 0.4 after about 25 cycles. An explanation for this decrease might be found in the very early occurrence of nanocrystalline grains for CoCrFeMnNi under tribological load compared to other fcc materials such as copper [3, 11]. Therefore, grain boundary sliding might play a substantial role in accommodating the shear stress and resulting in low friction coefficients [5]. Another reason could be a texture development by which $\left\{ {111} \right\}$ planes are re-oriented parallel to the surface, thereby simplifying dislocation slip [41], but this was not observed in the current results as discussed later. An increase in oxygen concentration within the wear track was determined after a 100 cycles experiment by means of EDS line scans (Fig. S2b and c). This is interpreted as tribo-oxidation starting between ten and 100 cycles. In this range, *µ* reaches its lowest value, which might, therefore, be the onset of tribo-oxidation. The increase in *µ* in region II is most likely due to an increase in surface oxidation. For compositional complex alloys, oxidation due to a tribological load was reported, but under less mild conditions [16, 42, 43]. When testing copper, tribo-oxidation was found to begin for a similar range of cycle numbers than CoCrFeMnNi under similar experimental conditions [35]. Within certain limits, the beginning of tribo-oxidation seems to be associated with the accumulated tribological load rather than with the specific materials in contact. This has to be further validated with more oxidation-resistant materials under similar conditions.

The change in friction behavior in region III after about 120 cycles is caused by the formation of wear particles. These are most likely a result of surface oxidation, leading to a surface embrittlement. As from now on a loop of oxidation, wear particle formation and again oxidation of the fresh surface is active, a higher variation in friction forces is measured. EDS analyses of the wear particles have shown a high amount of oxygen (Figure S2a). The average friction coefficient of *µ* = 0.63 after 120 cycles is in good agreement with the literature [17]. This might not be surprising, as the transferred material essentially leads to a self-mated contact in both cases. A quite similar progression of the friction characteristics with the duration of the experiment was reported by Blau for a dry sliding contact [44]. In agreement with Blau’s results, we also found wear particles next to the wear track after 1000 cycles, as indicated by a white arrow in Fig. 1c.

The slip traces next to wear track after 0.5 and one cycle in Fig. 1a, b give a first hint on a subsurface microstructure evolution. Dislocation nucleation and motion on a specific slip system lead to a reduction of local stresses on this slip system and the stresses on neighboring slip systems increase [13, 45]. As soon as dislocations on a specific slip system leave the material at the surface, parallel slip traces occur (Fig. 1a) [13]. The number of intersecting slip traces is equal to the number of active slip planes in the bulk material. Hence, in our samples, two or three slip planes are activated depending on the crystallographic orientation. We expect dislocation pileups underneath the slip traces in the bulk material. For cycle numbers exceeding 120, cracks are found in the subsurface material as a precursor to wear particle detachment [46].

### Microstructural evolution

When comparing the total thickness of the subsurface deformed layer for CoCrFeMnNi with that of other fcc metals like copper [3, 11], it becomes obvious that this layer is much thicker for the high-entropy alloy. Considering the higher Young’s modulus and yield strength due to solid solution hardening of CoCrFeMnNi, this is an unexpected result. In copper, after a single trace experiment under the exact same experimental conditions, a subsurface horizontal line of self-organized edge dislocations was found at a depth of 150 nm. This dislocation arrangement has the effective character of a small-angle grain boundary (the feature was referred to as dislocation trace line, DTL) [3]. The same feature was also observed for Ni, W, brass and Fe [11]. In contrast, for CoCrFeMnNi a fully developed tribo-material consisting of a fine-grained layer and a layer with tilted bands (Fig. 4a) develops already after a single trace. Differences between Cu and CoCrFeMnNi under nominally the same tribological load may result from different reasons. First, the frictional shear loading induces different plastic shear strains in these two materials. For the same testing parameters for copper, a plastic shear strain of 0.13 was estimated at the surface [12]. The grain boundary bending in Fig. 4a suggests a plastic shear strain of 5.3 at a depth of 1 µm below the surface. Most probably, this significantly higher shear strain in CoCrFeMnNi is due to the strong adhesive forces between CoCrFeMnNi and sapphire. According to simple geometrical considerations, more shear of the material is associated with a higher density of geometrically necessary dislocations. Keeping in mind that Cu and CoCrFeMnNi have similar lattice parameters (3.615 Å vs. 3.597 Å, respectively) and Burgers vectors, the dislocation density introduced by the tribological load in CoCrFeNiMn is expected to be higher than in Cu. The second reason for the microstructural differences is the SFE of the two materials, which is 41 mJ m^−2^ [47] for Cu and between 18 and 27 mJ m^−2^ [29] for CoCrFeMnNi. Based on the SFE, dislocations in Cu have a higher probability to cross-slip, making dislocation annihilation and rearrangement easier. The literature suggests dislocation cell formation through dislocation rearrangement under tribological load [3, 10]. Furthermore, planar slip and the formation of stacking faults as well as deformation twinning are favorable in CoCrFeMnNi at room temperature.

The total layer thickness for CoCrFeMnNi does not have a clear trend with increasing cycle number, while the one for Cu shows an increase [3]. Discrete dislocation dynamics simulations have shown an increase in dislocation density with each cycle [48]; therefore, an increase in the deformation layer thickness is expected. The reason for the pronounced difference in deformation layer thickness between Cu and CoCrFeMnNi might be the high work-hardening or the high initial plastic deformation of CoCrFeMnNi. All cross sections (Fig. 4a–c) show a fine-grained layer and a layer with tilted bands, indicating that the deformation mechanism is independent of the cycle number. As the fine-grained layer is thinner than the layer with tilted bands (Fig. 4d), the deformation mechanism leading to the fine grains seems to require a higher shear strain. Analyzing cross-sectional EBSD and TKD measurements, different regions of the altered microstructure, namely bands with localized dislocation movement, twins and nanocrystalline grains are observed.

### The formation of twins

The occurrence of twins under tribological load was mentioned in the literature for an austenitic steel [7]. For CoCrFeMnNi, deformation twinning was widely reported under different loading conditions [30, 49–52]. Twinning already occurred at lower strains compared to the maximum strain determined by grain boundary bending in Fig. 4a. It is conspicuous in Fig. 7a that in our experiments, the total layer thickness is higher when the twinned layer increases, while the thickness of the localized dislocation movement and the nanocrystalline layer remains almost constant. A possible reason for this behavior might be that the primary active slip plane is the same in all three layers. In Fig. 7b, the initial crystal orientations of the grains from which the TEM foils were cut are illustrated. Following the Venables twinning mechanism [53], twinning is active under a uniaxial tensile stress in grains with a crystallographic direction located at the right side of the inverse pole of the tensile axis. Under uniaxial compressive stress, only grains with a crystallographic direction on the left side of the inverse pole figure of the compression axis exhibit twinning in fcc materials.

All investigated grains with active deformation twinning have crystallographic orientations on the right side of the IPF in SD and ND. Based on the applied load, the material is compressed in ND as also shown in the schematic drawing in Fig. 9a. For this reason, the load in ND cannot be responsible for twinning. Hence, the tensile stress behind the sphere induced by the sphere’s motion is expected to be responsible for the formation of deformation twins under tribological load. As revealed in Fig. 2, we see significant evidence for strong adhesives forces between the sapphire sphere and CoCrFeMnNi, which could intensify the tensile stress in SD. The adhesion force was approximated to 0.05 N following the Johnson–Kendall–Roberts model with an average surface energy for metals (2 mJ m^−2^ [54]). Nevertheless, comparisons between the twinning process under uniaxial tensile stress and under tribological load are challenging, due to the complexity of the tribological stress field.

Characterizing a twin system as given in Fig. 8 allows to determine the orientation of the twin boundary within the crystal and the active twinning dislocation. The twin either forms under tensile or under compressive stress. Atomistic diagrams for both scenarios are presented in Fig. 9b, c. The twin in Fig. 9b can be formed under tensile stress in ND as a $[ {01\overline{1}} ]$ direction is aligned parallel to ND. At the same time, it remains unclear where tensile stress parallel to ND might originate from. For the twin in Fig. 9b to form, a tensile stress parallel to SD has to be present as indicated in Fig. 7b. Under uniaxial tensile load along SD such a twin cannot be formed because a $[ {100} ]$ direction is parallel to the tensile direction. Under tribological load however, the stress field is much more complex and it might lift this restriction. As this twin elongates along the original lattice in ND direction, it can only have formed behind the sphere. It is unlikely that the twinning accommodates a straining of the lattice in the opposite direction of the normal load. For the formation of the second geometrically possible twin in Fig. 9c, a compressive load parallel to SD or a tensile load parallel to ND is conceivable. The crystal orientation of the given system would allow both of these, considering a uniaxial load. A tensile stress parallel to ND is not operative under the tribological load as discussed above, whereas a compressive stress parallel to SD occurs in front of the sphere. The consideration of the two geometrically possible twins demonstrates that the stress parallel to SD is probably decisive for twin nucleation. The stress parallel to ND is negligible. This interpretation is supported by the RSS calculations after Hertz [31] and Hamilton [34] given in Fig. 9d and e, respectively. Only the calculation according to Hamilton reaches the critical resolved shear stress (CRSS) for twinning in front of the sphere, which was determined to be between 110 MPa [49] and 235 MPa [30] for CoCrFeMnNi. Comparing the geometrically possible twins in Fig. 9b, c with the RSS calculations, most probably the twin in Fig. 9c nucleates under compressive stress parallel to SD in front of the sphere. This result seems contradictory to the before discussed original crystal orientation, whereby tensile stresses parallel to SD behind the sphere are responsible for twinning. The difference is that the real twinning system in Fig. 8 was analyzed close to the surface, where the crystal is rotated in contrast to the original crystal orientation presented in Fig. 7. However, the question where exactly the twin starts to form cannot be answered with full certainty. In case twins were to nucleate in front of the sphere, the crystal has to exhibit a large rotation before twins form. The stress field behind the sphere then would not influence the microstructural evolution at all. This seems unlikely, especially as the stress field’s magnitude in front and behind the sphere is comparable, while the stress field’s spacial dimension is much larger behind the sphere.

In Fig. S3a, one twin lamella (marked with a black arrow) partly exhibits high-angle grain boundary character to its adjacent grain. This is strong evidence for the activation of both dislocation slip and deformation twinning in the twinned region as twin dislocation interactions can destroy the coherency of a twin boundary. At the same time, this process suggests that after twinning significant dislocation glide occurs for the given crystal orientation. Interestingly, it is this twin in question which is thicker compared to the other twin detected in the same TEM foil. This could be an indication for a stress localization, an inhomogeneous twin formation as this could be caused by local changes in the SFE or short range ordering.

With the cross-sectional STEM images in Fig. 5a it is likely that already during the very first trace, the future fate of the subsurface material is determined. There is virtually no difference between subsurface microstructures after 0.5 and one cycle (Fig. 5a, b). In case of a grain rotation such that it deforms by dislocation motion during the trace and by twinning during the retrace, a significant difference would occur. This was not observed.

### Localized dislocation movement

The bulk material and the twinned layer are separated by a region dominated by localized dislocation motion (LDM); see the yellow lines in Figs. 6b, c and S3. Generally, LDM is caused by planar slip due to low and medium SFE, high concentrations of solute atoms and/or short range ordering [55]. For CoCrFeMnNi, both a medium SFE [29, 56] as well as a high concentration of solute atoms are satisfied of which both are not present in copper [3]. Since copper exhibits a higher SFE, either this or the solute content in CoCrFeMnNi remains a probable reason for the confined dislocation motion. The thickness of the layer is almost constant for all TEM foils investigated and the layer moves deeper into the subsurface material as the twinned layer gets thicker (Fig. 7a). Although the responsible dislocations for dislocation slip and twinning differ, it is remarkable that under tribological load both—twinning and octahedral slip—show the same depth dependency concerning grain orientation. For the dislocations to move within the LDM layer, the decisive factor is the RSS. According to Hamilton, the shear stresses in the plane determined by ND and SD are higher the closer to the surface. We therefore propose that twinning and octahedral slip take place on a similar glide plane, whereby the crystallographic orientation is responsible for the RSS depth, which has to exceed 110–235 MPa for twinning and 78–82 MPa for dislocation glide in CoCrFeMnNi [30, 49]. At a certain depth, the RSS is no longer sufficient for the activation of deformation twinning and the material responds by accommodating the shear load through the localized movement of dislocations within this LDM-zone. The localization of dislocation motion was previously reported for CoCrFeMnNi at low tensile strain [28, 50].

As the thickness of the LDM layer is approximately constant for all grains investigated, we infer that the decrease in the dominant shear stress below the twinned layer with increasing depth is grain orientation independent. However, LDM is also observed on secondary and tertiary slip systems in some cases depending on the grain orientation, forming chessboard-like patterns (see Fig. 6b). Such patterns are more likely to be observed closer to the surface, as higher stresses are necessary to activate additional slip systems with presumably lower RSS than the primary. Since these slip systems provide non-parallel slip planes, the probability of complex dislocation interactions potentially resulting in sessile dislocation segments is enhanced and significant strain hardening is expected [8, 45]. As a consequence, the further increase in the total deformation layer thickness should be less than in materials not possessing such an elementary deformation mechanism. This is exactly what we observe when comparing the dependence of the deformation layers thickness between copper [3] and CoCrFeMnNi as a function of cycle number.

In addition, the LDM leads to distinct slip traces on the surface. When closely examining the surface SEM images presented in Fig. 1a, we find a maximum of three slip planes were activated which is demonstrated by three different slip traces orientations on the sample surface (Fig. 1a).

Underneath the wear track, dislocations cannot leave the sample at the surface, as they are stopped at the transition between twinned and nanocrystalline layer. As soon as a potential dislocation pileup is large enough, the applied stresses cannot further activate dislocation motion on the slip plane of the pileup and parallel slip might be activated. This mechanism was previously suggested, when similar lines were observed in austenitic steel under a tensile stress [45] and for tribological load [13].

In most cases, the bands of LDM are tilted toward the direction of the initial trace of the sapphire sphere (see Figs. 6b, c and S3). This may be explained as follows: the tilt is based on a rotation of the entire grain and the slip system with the highest RSS is always tilted in this direction, or this is the orientation that dislocations can follow the sliding sphere on during the first pass of the sphere the easiest [48].

No differences could be found when comparing LDM between CoCrFeMnNi and classical alloys with a comparable SFE such as austenitic steels [7, 8]. At the same time, dislocation glide in HEAs may further be influenced by the strong lattice distortion [15] and changes in dislocation dissociation by local variations in the SFE [57].

Mediated by the very localized dislocation motion within the LDM-layer, rotations of the crystallographic orientation are induced as they are visualized by the point-to-point misorientation along the white arrow in Fig. 6c and plotted in Fig. 6d. An increase in misorientation in reference to the starting point is interpreted as a forward rotation and a decrease in misorientation as a backward rotation. We speculate that the first LDM band leads to a forward rotation. Next, the local stress field changes due to the lattice rotation and, consequently, the subsequent LDM band is generated such that it compensates the distortion field of the first crystal rotation, resulting in a backward rotation. The point-to-point misorientation shows that the LDM leads to a maximum misorientation of 2° at the chosen location. The literature reports similar alternating rotations under a tribological load in a bronze [9]. In this case, planar slip is as likely as in CoCrFeMnNi. As LDM bands were not observed in pure copper [3, 10], in which dislocations exhibit wavy slip, LDM might be attributed to being an elementary mechanism only in medium to low SFE materials. Leading to the conclusion, that planar slip prevents homogenous crystal rotation under tribological load.

Plotting the depth-dependent grain orientation in inverse pole figures as in Fig. 10 yields that ND directions have to rotate toward $[ {111} ]$ to conform with the texture evolution mentioned in the literature [41, 58]. For our experiments however, $\langle {001} \rangle$ directions align parallel to ND with decreasing distance to the surface. This does not correspond to a compression texture [59], as it might have resulted from the applied normal load. In case of a dominant tensile stress along SD, a [112] direction is expected to align parallel to SD with decreasing distance to the surface [59]. Under a tribological load, it seems like a direction of the bisector between $[ {001} ]$ and $[ {011} ]$ is aligned parallel to the SD. Why exactly this rotation in ND and SD is observed is not entirely clear yet. It is however not easily explained with a simplified calculation of the stress field under the moving sphere. The inverse pole figure in TD (Fig. 10c) shows the smallest rotation in comparison to SD and ND, indicating that the crystal rotation axis is close to TD.

### Nanocrystalline layer

When subjecting CoCrFeMnNi to high pressure torsion (HPT) experiments, the development of a nanocrystalline saturation microstructure with about 50 nm in grain size was reported [51]. This is close to the nanocrystalline grains observed in Fig. 5. For HPT, this grain size was achieved for a shear strain between 4 and 50 [51]. Estimating the shear strain in our experiments after half a cycle based on the bending of a grain boundary (see dashed line in Fig. 4a) yields similar shear strain values. In addition, Hamilton’s solution [34] predicts the highest shear stresses to act directly at the sliding interface (for experimentally determined friction coefficients higher than 0.3, as observed in the present case). The most pronounced nanocrystalline layer was found in the sample that exhibited the thickest twinned layer (depicted in blue color in Fig. 7), indicating that twinning seems to be a contributing or facilitating mechanism during the microstructural fragmentation process. It was previously shown that this is the case for some medium SFE materials [51, 60]. The same holds true for HPT experiments with CoCrFeMnNi [51]. In case of SFE being higher, dislocation rearrangement and significant dynamic recovery is expected [3, 10]. The development of a nanocrystalline layer can be also explained by shear band formation parallel to the surface [7].

Transfer of CoCrFeMnNi to the counter body and following re-deposition processes might also play a substantial role during the formation of nanocrystalline grains. Nevertheless, we have no information about how long an individual patch of transferred CoCrFeMnNi sticks to the sapphire sphere and when re-deposition exactly occurs. This duration might significantly affect the imposed deformation on the transferred material in the tribo-contact. During imaging the sapphire sphere (Fig. 2b), the grain size within the transferred HEA could not be resolved, but the same grain size as in the nanocrystalline layer is expected. As the sliding speed was chosen deliberately slow, we expect that frictional heating did not caused any recrystallization during our experiments. Furthermore, it was suggested [12] that several sliding cycles are needed for dynamic recrystallization, even at higher sliding velocities, leading to a decreasing nanocrystalline layer. Such a trend could not be found in the present work. Recent results have demonstrated that alloying may suppress grain growth during tribological loading, stabilizing a nanocrystalline microstructure and allowing for extremely low friction and wear [5]. A friction coefficient for a dry sliding contact of below 0.5 was explained by means of the dominance of grain boundary sliding over dislocation motion. In the results presented here, a friction coefficient below 0.5 was obtained between 10 and 100 cycles (Fig. 3). For exactly these cycle numbers, the subsurface microstructure exhibits the thickest fine-grained layers (Fig. 4d).

### Oxygen-rich layers after 1000 cycles

When investigating the chemical composition of a wear track’s cross-sectional area after 1000 cycles by EDS (Fig. 11), we found that positions of oxygen enrichment coincide with bright contrast in SEM (Fig. 5c). In the literature, oxidation of surface-near regions under tribological loading has been widely reported [35–37]. In contrast to the present layered structure in CoCrFeMnNi, oxygen-rich hemispherical clusters were found beneath the surface for pure copper [35]. These layers found for CoCrFeMnNi are likely caused by mechanical mixing subsequent to an initial oxidation at the surface. This is followed by material transfer to the sapphire sphere and the continued oxidation of the freshly exposed CoCrFeMnNi. As sliding progresses, material is re-deposited on the oxidized wear track. The repetition of these processes results in the observed, multilayered structure. When deliberately starting with metallic multilayers, several authors report intermixing by vortex formation [4] and shear instabilities [61, 62]. The nearly perfect parallel alignment of the oxygen rich bands may hint to shear localization facilitating oxygen diffusion. Stress concentrations in these presumably more brittle regions might be the origin of wear particles.

## Conclusions

We investigated the frictional response of CoCrFeMnNi in a dry reciprocating contact against a sapphire sphere under mild tribological load. We focused on the elementary mechanisms accommodating the shear load and being active in the subsurface material. By systematic SEM, STEM, TKD and EDS analyses, we find that:

- CoCrFeMnNi deforms very similar to other medium SFE metals and alloys. No HEA-specific deformation mechanisms were identified.
- Already after only a single trace, the subsurface material can be divided into three separate layers. From the contact surface, these are: (1) a nanocrystalline, (2) a twinned and (3) a layer of localized dislocation movement.
- The nanocrystalline layer is formed by intense shear deformation of the material being closest to the surface and is most probably assisted by deformation twin fragmentation. Grain boundary sliding might be responsible for accommodating the high strains at and very near the surface.
- In the twinned layer, we find the same original crystallographic orientations to twin during deformation as expected under uniaxial tensile stress along the SD behind the sphere.
- The analysis of an active twin system and calculating the resolved shear stresses suggest that the twin formed under a compressive stress parallel to SD; thus in front of the passing sphere. All analysis points to stresses along SD being responsible for twinning under a tribological load. The apparent contradiction to the point above can as of now not yet be resolved.
- The thickness of the twinned layer determines the combined thickness of all three layers and is most probably determined by the crystallographic orientation. This might suggest that twinning and octahedral slip take place on a similar glide plane, whereby the crystallographic orientation is responsible for the RSS depth.
- Unexpectedly, high adhesive forces act between the sapphire counter body and the CoCrFeMnNi alloy. These seem to alter the stress state and may be responsible for the early onset of severe subsurface plastic deformation.
- Within the zone of localized dislocation movement, very localized plastic deformation caused by planar slip results in slip traces protruding from the wear track surface. Some of these bands mediate a forward and others a backward rotation.
- An unexpected texture development was identified. For high strains, i.e., close to the surface, the crystal system rotates toward $\langle {001} \rangle$ in ND and toward the bisector connecting $\langle {001} \rangle$ and $\langle {011} \rangle$ in SD. This is not compatible to either tensile or compressive texture evolution, nor to textures under tribological load as they are reported in literature.
- Beyond 120 sliding cycles, the material demonstrates first signs of tribologically induced oxidation. After 1000 cycles, a layered structure with alternating regions of oxygen depletion and enrichment is identified. This horizontal band-like structure most probably originates from material transfer between the CoCrFeMnNi plate and the counter body.

## Electronic supplementary material

Below is the link to the electronic supplementary material.

Supplementary file1 (DOCX 6461 kb)
