# Supplementary material for: Microstructural changes in CoCrFeMnNi under mild tribological load
Source: J Mater Sci. 2020 May 27;55(26):12353–72. doi: 10.1007/s10853-020-04806-0 (PMC7311379; doi:10.1007/s10853-020-04806-0)

Microstructural changes in CoCrFeMnNi under mild tribological load

Supplementary Information

Antje Dollmann^†,‡^, Alexander Kauffmann^†^, Martin Heilmaier^†^, Christian Haug^†,‡^, Christian Greiner^*†,‡^

^†^ Institute for Applied Materials (IAM), Karlsruhe Institute of Technology (KIT),

Kaiserstrasse 12, 76131 Karlsruhe, Germany

^‡^ KIT IAM-CMS MicroTribology Center (µTC), Strasse am Forum 5, 76131 Karlsruhe, Germany

*To whom correspondence should be addressed: greiner@kit.edu

**Figure S1. Top and cross-sectional perspective of an unloaded surface.** a) backscatter electron image of the top view; b) scanning electron microscope image; and c) scanning transmission electron image of the cross-section.


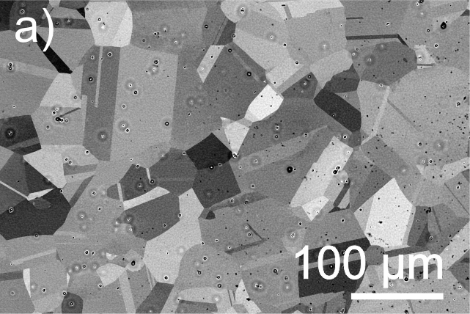

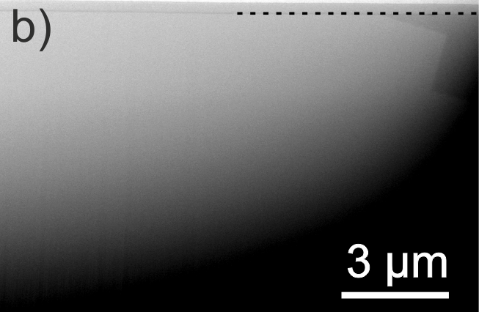


| 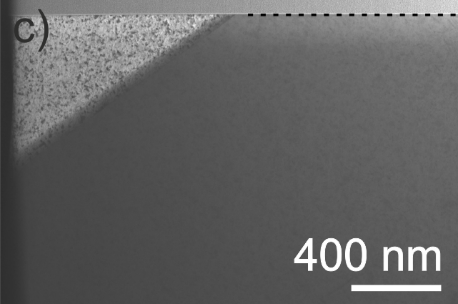 |
| --- |
|  |

**Figure S2. EDS measurements of wear particles and wear tracks.** a) Elemental composition of wear particles; b) oxygen concentration along a line scan in the center of the wear track perpendicular to the sliding direction (SD) after 10 cycles, c) after 100 cycles and d) after 500 cycles. The dashed lines in b), c) and d) mark the edges of the wear track.


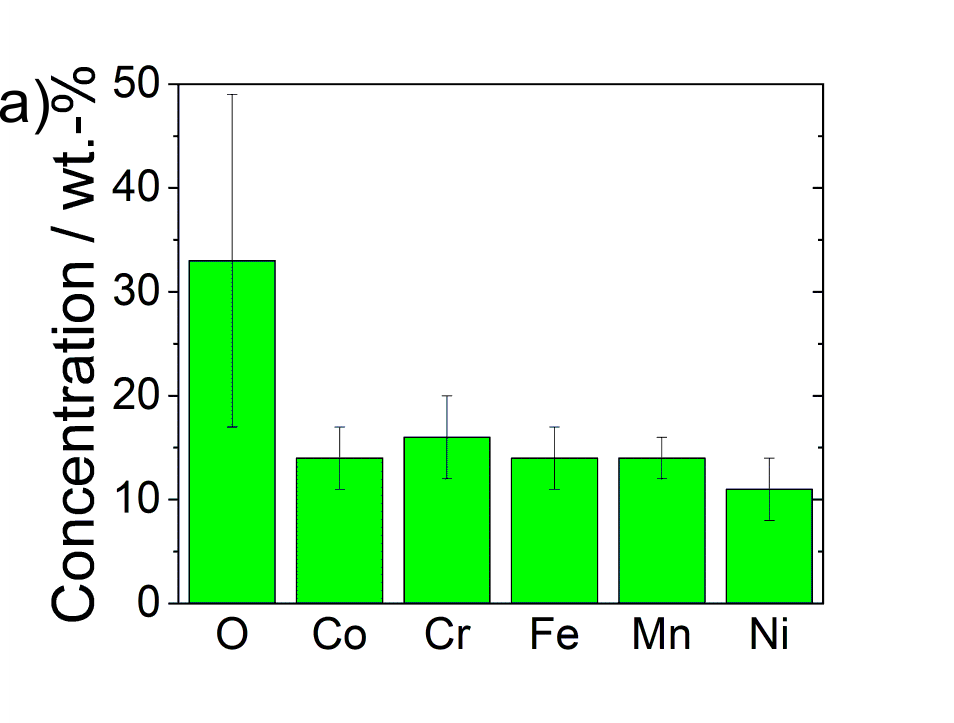

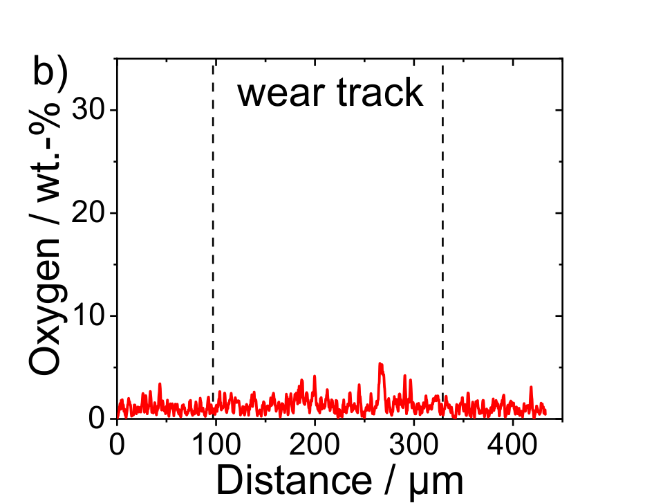


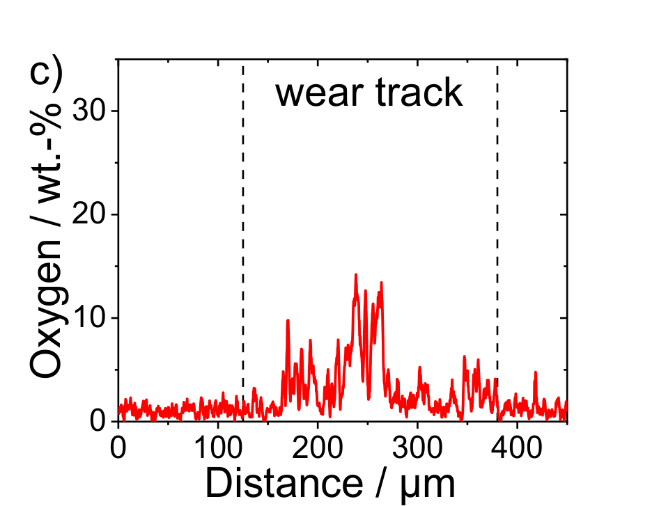


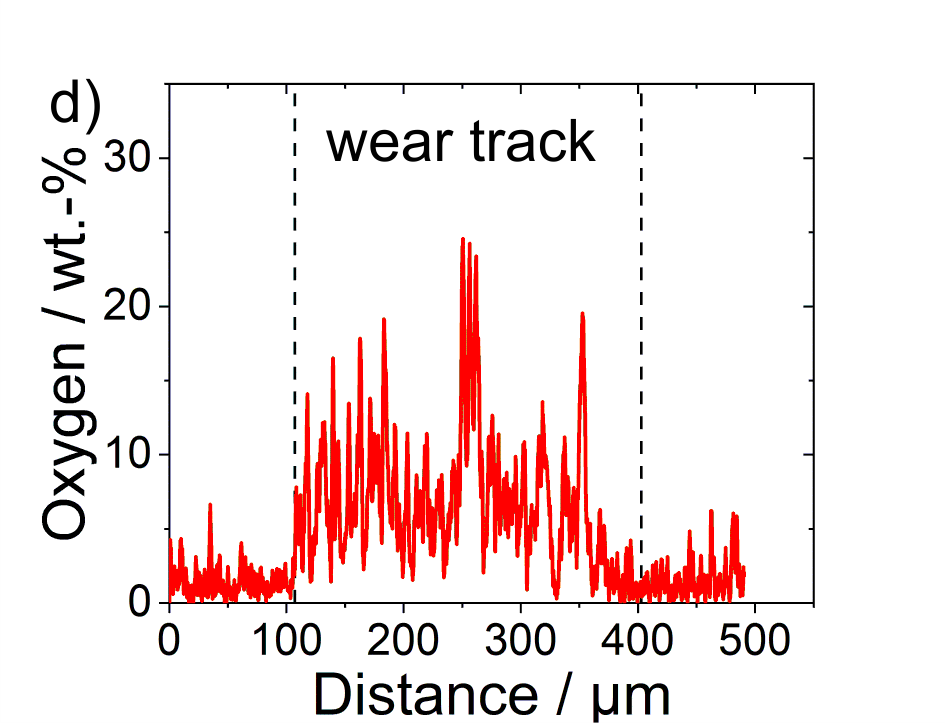


**Figure S3. Overview of all Transmission Kikuchi Diffraction (TKD) and cross-sectional EBSD measurements.** TKD measurements are shown in the first and third column and the cross-sectional EBSD measurements in the second and fourth column. The results are given in normal direction (first and second column) and as Kernel Average Misorientation (KAM) maps (third and fourth column). The coloring of a), b) and c) is the same as in Fig. 7. a) and b) is after 0.5 cycles; c) after one cycle; and d) after 1000 cycles. The TEM-foils were prepared in the center of the wear track, along the sliding direction (SD). SD is from left to right.


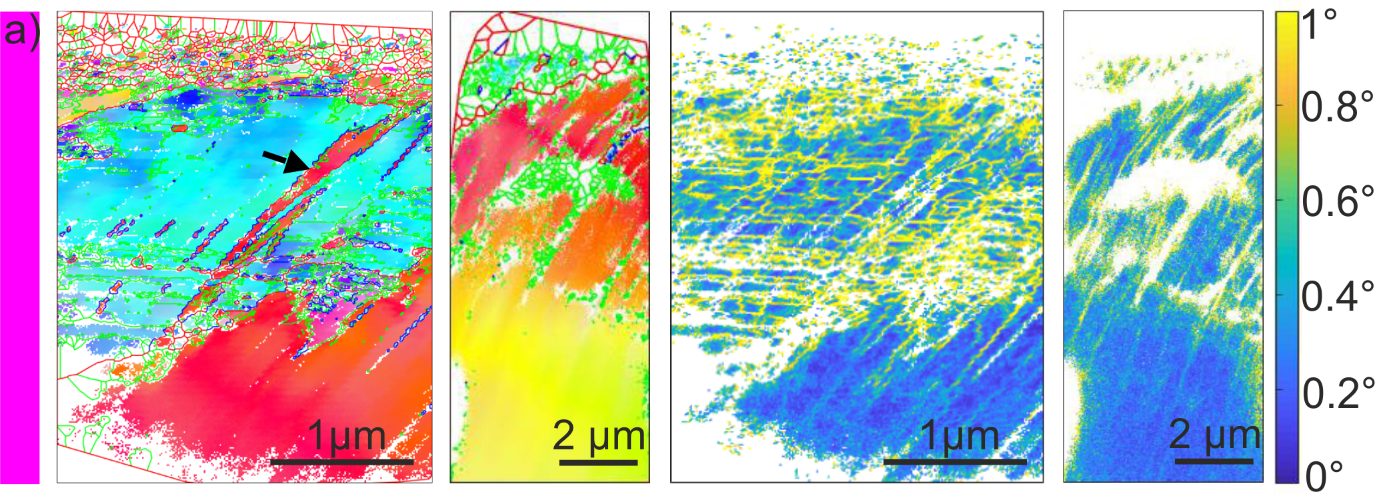


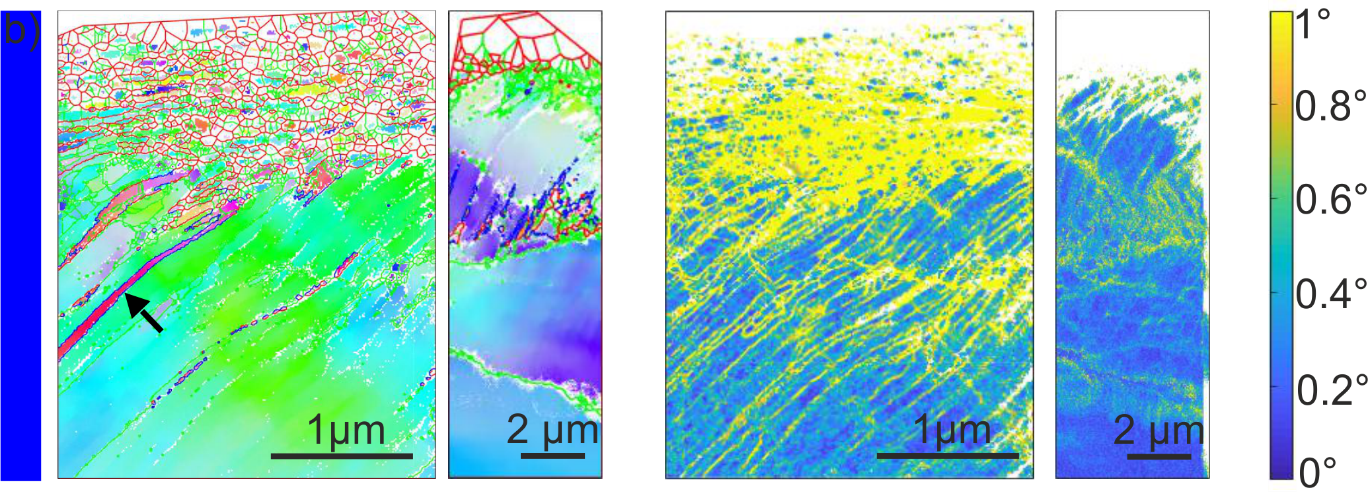


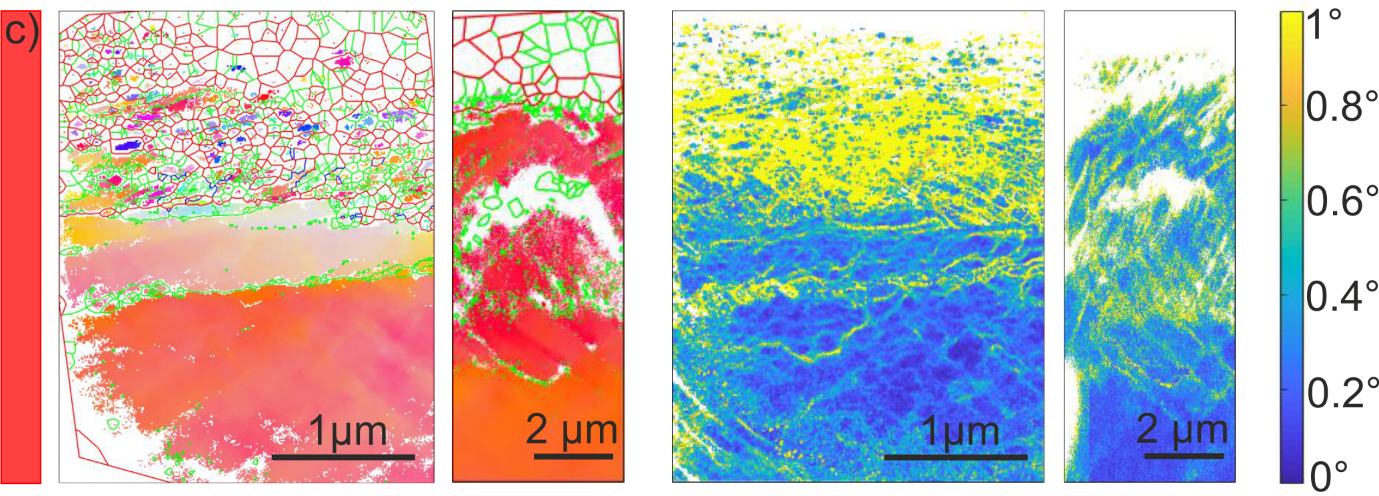

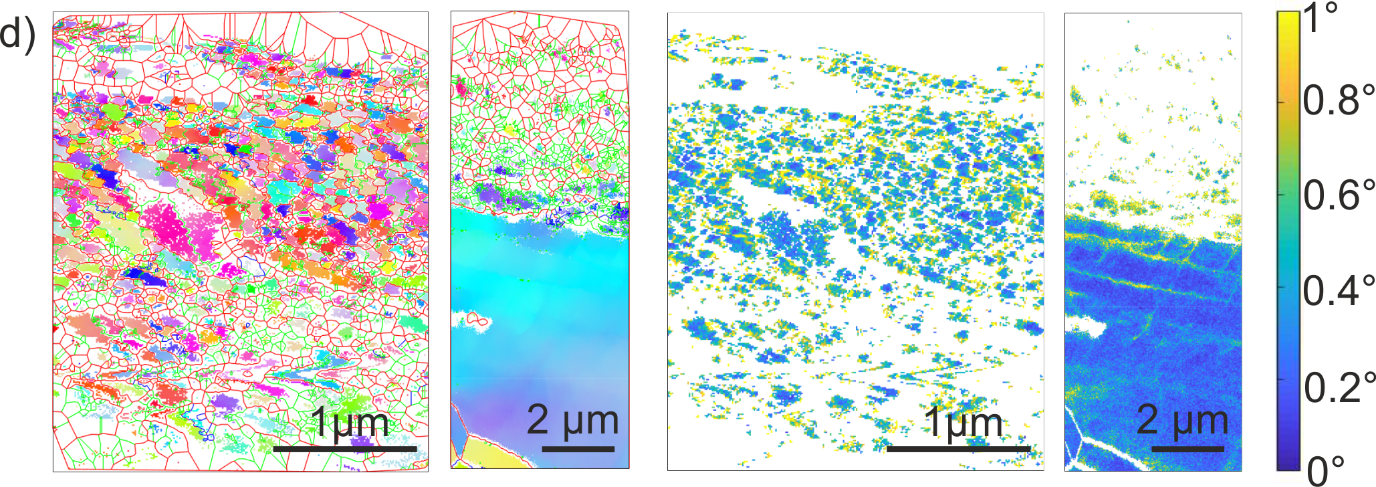

Supplement: Supplementary file 1 — Supplementary file1 (DOCX 6461 kb) [file 10853_2020_4806_MOESM1_ESM.docx]
